# Pathogenic analysis of *Borrelia garinii* strain SZ isolated from northeastern China

**DOI:** 10.1186/1756-3305-6-177

**Published:** 2013-06-17

**Authors:** Qiong Wu, Zhijie Liu, Jidong Wang, Youquan Li, Guiquan Guan, Jifei Yang, Ze Chen, Jianxun Luo, Hong Yin

**Affiliations:** 1 State Key Laboratory of Veterinary Etiological Biology, Key Laboratory of Veterinary Parasitology of Gansu Province, Key Laboratory of Grazing Animal Diseases MOA, Lanzhou Veterinary Research Institute, Chinese Academy of Agricultural Science, Lanzhou 730046, China

**Keywords:** *B. burgdorferi*, Pathogenicity, Kinetics of spirochete, Disease severity

## Abstract

**Background:**

Various genospecies of *Borrelia burgdorferi* sensu lato (s.l.) have been identified from patients and animals worldwide. Genospecies-related dissemination of disease has been reported. The present study aimed to elucidate the pathogenicity of infections caused by *B. garinii* SZ isolated in China. *B. burgdorferi* B31 and *B. afzelii* BO23 were used for comparison*.*

**Methods:**

Spirochete load in blood and tissue samples of infected mice were measured by minor groove binder-based real-time polymerase chain reaction. The kinetics of spirochete dissemination and disease severity were assessed in BALB/c mice.

**Results:**

The pattern of bacterial load differed between the three genospecies. The *B. garinii* SZ strain is highly pathogenic and can trigger multi-system pathological damage in mice.

**Conclusions:**

Spirochete dissemination, persistence, tissue tropism and disease severity varied significantly, suggesting that different genospecies may play an important role in the pathogenicity and development of clinical diseases.

## Background

The *Borrelia burgdorferi* s.l. complex comprises 18 known *Borrelia* genotypes. Three of them predominate as human pathogens: *B. burgdorferi* sensu stricto (s.s.), *B. garinii* (including *B. garinii* OspA type 4, recently designated *B. bavariensis*) and *B. afzelii*[1]. However, *B. valaisiana*, *B. lusitaniae*, *B. spielmanii* and *B. bissettii*, have been also occasionally isolated or detected in patient specimens [2,3]. The symptoms and severity of the disease vary in *B. burgdorferi* genospecies. *B. garinii* is primarily associated with neuroborreliosis [4], *B. afzelii* with acrodermatitis chronic athrophicans [5] and *B. burgdorferi* s.s is prevalent in Lyme arthritis [6].

Examination of the relationship between genospecies and clinical symptoms using real-time polymerase chain reaction (PCR) to detect *B. burgdorferi* levels in all tissues of C3H/HeJ mice and BALB/c mice reported a positive correlation between clinical symptoms (arthritis) and spirochete burden [7]. The correlation of *B. burgdorferi* genospecies with clinical presentation is potentially valuable in explaining the disease manifestations of Lyme borreliosis [8]. Numerous new isolates of *B. burgdorferi* have been obtained. However, the pathogenicity of these isolates is unclear.

*B. garinii* is the main genotype found in China [9]. To elucidate the pathogenicity of the *B. garinii* SZ isolated in China, the kinetics of spirochete dissemination and the severity of the disease were evaluated in a murine model. Considering that different genotypes of *B. burgdorferi* could potentially affect disease pathogenicity, *B. burgdorferi* B31 and *B. afzelii* BO23 were used for comparison. Dissemination of spirochetes in blood and tissues was evaluated using TaqMan™ minor groove binder (MGB) - real time polymerase chain reaction (PCR) and disease severity was assessed by histopathologic assessment of inflammatory cell infiltrates and the extent of tissue necrosis.

## Methods

### Bacterial strains

*B. burgdorferi* B31 and *B. afzelii* BO23 were purchased from ATCC (Manassas, VA) and were passaged five times *in vitro*. *B. garinii* SZ was isolated from *Dermacentor* ticks collected in Shangzhi County of Heilongjiang Province, China [10]. These strains were incubated in BSK-H medium at 33°C and observed with a dark-field microscope every other day. The bacteria were harvested by centrifugation at 5,000 g when they reached logarithmic phase and were washed twice with phosphate buffered saline (PBS). The suspension was adjusted to a density containing 10^5^ cells/ml using a Petroff-Hausser counting chamber.

### Mice and infection

Female specific pathogen-free BALB/c mice were obtained from the Breeding Laboratory, Lanzhou Veterinary Research Institute (Lanzhou, China). All mice were 4 weeks old at the time of infection. The mice were divided randomly into control, SZ, BO23 and B31 groups each consisting of 25 mice. Mice in the experimental groups received an intraperitoneal injection of 200 μl PBS containing 10^5^/ml of *B. burgdorferi* cells in experimental groups. Control mice were injected with 200 μl PBS. Tissue specimens from blood, brain, tongue, heart, lung, liver, spleen, kidney, lymph, bladder, joints and skin were collected on days 2, 5, 9, 15, 30, 60, 90 and 150 after infection.

### DNA preparation

DNA was prepared using the QiaAmp tissue kit and Puregene blood core kit (Qiagen, Valencia, CA) following the manufacturer’s instructions. The DNA was finally eluted in 200 μl distilled water and stored at −20°C until use.

### Real-time PCR

Simultaneous detection and quantification of *B. burgdorferi* DNA were performed in a model MX3000 P real-time PCR machine (Stratagene, La Jolla, CA). The targeting gene was the *fla*B single copy gene [11]. The TaqMan™ MGB-probe and corresponding primers were designed using Primer Express™ software (Applied Biosystems, Foster City, CA). The upstream primer was 5′-GTG CAT TTG GTT ATA TTG AG-3′, the downstream primer was 5′-CAG ACA GAG GTT CTA TAC A-3′ and the probe was FAM-5′AAT AGA GCA ACT TAC AGA-3′-MGB. The probe was purchased from Applied Biosystems and the primers were from Shanghai Shenggong (Shanghai, China).

For quantitation of the mouse host, *β-actin* was chosen. The upstream primer was 5′-AGA GGG AAA TCG TGC GTG AC-3′, the downstream primer was 5′-CAA TAG TGA TGA CCT GGC CGT-3′ and the TaqMan™ probe was FAM-5′CAC GGC CGC ATC CTC TTC TTC C-BHQ1-3′.

The plasmids containing the *fla*B gene of *B. burgdorferi* B31 and mouse *β-actin* gene served as standards. The plasmid containing 1100 bp *flaB* gene was obtained by PCR amplification using the *fla*B upstream primer 5′-ATG ATT ATC AAT CAT AAT ACA TCA-3′ and downstream primer 5′-TTA TCT AAG CAA TGA CAA-3′. The PCR fragment was cloned into the pGEM-T vector (Promega, Madison, WI) and then propagated in competent *Escherichia coli* JM109 (TaKaRa, Dalian, China). The plasmid containing the 129 bp *β-actin* gene was obtained by PCR amplification using the upstream primer 5′-AGA GGG AAA TCG TGC GTG AC-3′ and the downstream primer 5′-CAA TAG TGA TGA CCT GGC CGT-3′ and cloned as described above. Plasmid DNA was quantified using a model 2000 spectrophotometer (NanoDrop Technologies, Wilmington, DE). The plasmid control in the real-time PCR was to ensure the efficacy of the assay and to compile standard curves for determination of the *fla*B copy number in mouse tissue. Ten-fold serial plasmid dilutions were prepared, ranging from 10^0^-10^7^ copies.

The PCR mixture (25 μl total volume) consisted of 300 nM primer, 200 nM probe, 200 nM deoxynucleoside triphosphates, 3.5 mM MgCl_2_, 2 μl DNA, 1 U AmpliTaq Gold and 1 × PCR buffer. Amplification and detection were performed using 40 cycles 95°C for 10 s, 57°C for 30 s (*fla*B) and 95°C for 10 s and 60°C for 30 s (*β-actin*). Actin and *fla*B PCR used separate reaction mixtures. The FAM signal was standardized to the passive reference ROX, which was included in the reaction buffer. Individual samples were run in triplicate.

### Histopathologic analysis

On days 30, 60 and 90 after infection, three mice in each group (total 12) were sacrificed. Brain, heart, lung, liver, kidney, spleen and joint tissues were obtained and immersed in formalin. Tissues were embedded in paraffin, sectioned at a thickness of 5 μm and stained with hematoxylin and eosin (H&E). Each histopathologic parameter was assessed and scored separately by two pathologists using a semi-quantitative criteria-based scoring method [12]. Scores of 0 (absent,-), 0–10 (mild,+), 10–20 (moderate,++) and 20–30 (severe,+++) were assigned to each parameter, and the average scores were used to reflect the overall severity for each group of mice.

### Statistical analysis

Quantitative data obtained by real-time PCR were analyzed using Prism and Excel 97 software. Significant differences were determined by unpaired Student’s t test implemented in Prism software, with p <0.05 considered statistically significant.

### Ethical approval

The animal experiments in this research were approved by Gansu Provincial Science and Technology department in China and in accordance with the Animal House of Lanzhou Veterinary Research Institute Instructions. The license No: SYXK2010-0001.

## Results

### Detection and quantification of *B. burgdorferi* in mouse tissues

**Figure 1:**
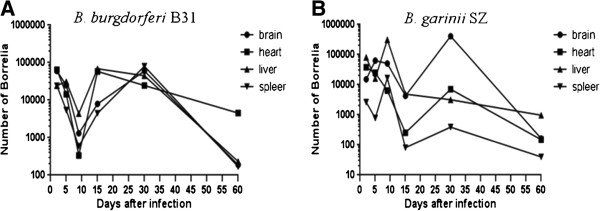
**Kinetic distribution of spirochetes in tissues of mice.** BALB/c mice were sacrificed at various time points after infection. The brain, heart, liver and spleen tissues were tested for Borrelia by qPCR. (**A**): *B. burgdorferi* B31 and (**B**): *B. garinii* SZ. The mean numbers of spirochetes per 10^6^ mouse cells (calculated on the basis of*β-actin* standard curves) are given.

Two microliters of mouse DNA or external standard template containing 10^0^-10^7^ copies of *B. burgdorferi fla*B were used. Therefore, the theoretical number of bacteria in our sample was 100 times lower. All mice infected with B31 and SZ became *B. burgdorferi* positive, whereas *B. afzelii* BO23 failed to establish an infection. Spirochetes in the blood, brain, tongue, heart, lung, liver, spleen, kidney, lymph, bladder, joint and skin tissues were collected from mice infected with *B. burgdorferi* B31, *B. afzelii* BO23 and *B. garinii* SZ 2, 5, 9, 15, 30, 60, 90 and 150 days after infection (Additional file 1: Table S1 and Table 1). Spirochete DNA was detected by qPCR in all tissues according to time course during infection; the overall patterns of bacterial load followed a fluctuated trend among the isolates during the 30 days after infection. For mice infected with *B. burgdorferi* B31, a relatively higher spirochete burden in tissues was reached at 2 days after infection, peaking on day 5, gradually subsiding from days 5 to 9 and then gradually recovering from day 15 to day 30 (Figure 1A). *B. garinii* SZ numbers were greater at day 5, peaked at day 9, and gradually subsided through day 15 (Figure 1B). On day 30 after infection, *B. burgdorferi* B31 produced the more consistent and higher spirochete burden than SZ. Sixty days after infection, the differences in the numbers of spirochetes between tissues from the two *Borrelia* genospecies strains became smaller but the persistent tissue tropism was significantly different. On days 90 and 150 after infection, persistence of *Borrelia* was evident in spleens of B31 infected mice and in livers of SZ infected mice (Additional file 1: Table S1). The number of spirochetes in blood was lower compared with other tissues at the indicated times (Table 1).

**Table 1:** **Spirochete burden in blood infected by different strains at different times post-infection (p.i.)**^**a**^

**Time p.i**	**B31**	**SZ**	**BO23**	**Control**
2 d	263	153	278	0
5 d	329	105	558	0
9 d	1260	160	1060	0
15 d	258	875	250	0
30 d	366	759	136	0
60 d	144	129	89	0
90 d	0	0	0	0

^a^ BALB/c mice were sacrificed at various time points after infection. The blood was tested for Borrelia by qPCR. The mean numbers of spirochetes per 10^6^ mouse cells (calculated on the basis of *β-actin* standard curves) are given. days (d).

### Severity of disease assessed by histopathologic analysis

**Figure 2:**
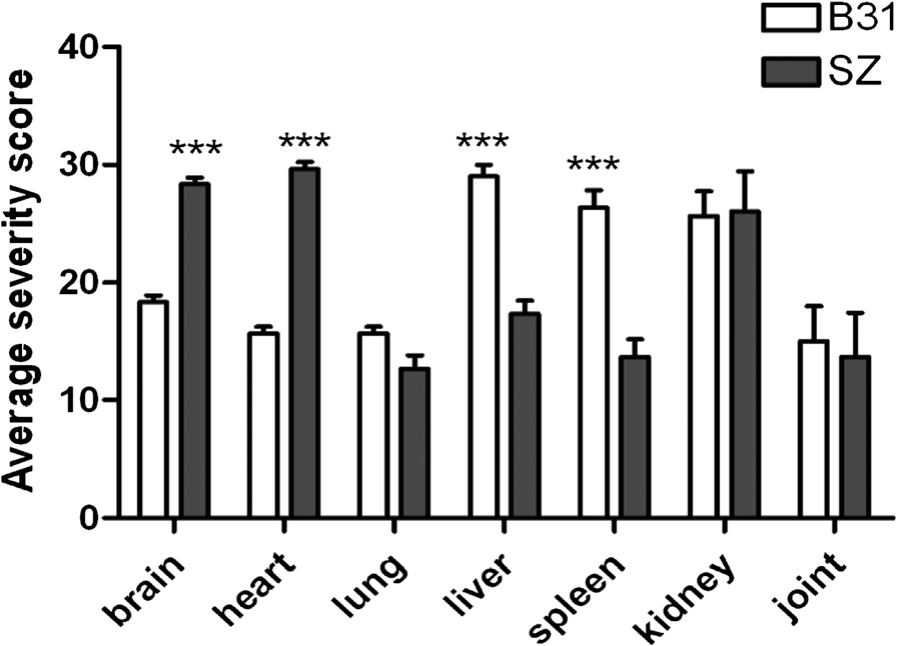
**Severity of tissue damage caused by different *Borrelia *strains 30 days post infection.** Each histopathologic parameter was assessed and scored separately by a semi-quantitative criteria-based scoring method [12]. The average scores were used to reflect the overall severity for each group of mice. The differences in disease severity between B31 and SZ infected mice were significant in brain, heart, liver and spleen tissues (p < 0.05). The asterisks indicate samples whose values are statistically significantly different between B31 and SZ (*, P <0.05; **, P <0.01; ***, P <0.001). Error bars indicate standard deviations.

Histopathology of mice infected with *B. burgdorferi* B31, *B. garinii* SZ and *B. afzelii* BO23 on days 30, 60 and 90 after infection was studied. The differences in disease severity between B31 and SZ infected mice were significant in brain, heart, liver and spleen tissues (p < 0.05) 30 days after infection (Table 2, Figure 2). No significant pathologic abnormality was observed in BO23 infected mice and no pathologic abnormality was observed in any tissues of control mice.

**Table 2:** **Severity of tissue damage caused by different *Borrelia *strains **^**a**^

	**Day 30 after infection**			**Day 60 after infection**		**Day 90 after infection**		
	**B31**	**SZ**	**BO23**	**B31**	**SZ**	**B31**	**SZ**	**Control**
Brain	++	+++	—	++	++	+	++	—
Heart	++	+++	—	++	++	+	++	—
Lung	++	++	—	+	+	+	+	—
Liver	+++	++	—	+++	++	+++	+	—
Spleen	+++	++	—	++	++	++	++	—
Kidney	+++	+++	—	+++	++	++	+	—
Joint	++	++	—	+	+	+	—	—

^a^ +++ severe, ++ moderate, + mild, —absent.

Three mice in each group were sacrificed at 30, 60, and 90 days after inoculation. Disease severity was assessed by histopathologic assessment of inflammatory cell infiltrates and necrosis extent in tissues. The differences in disease severity between B31 and SZ infected mice were significant in brain, heart, liver and spleen tissues (P <0.05) at 30 days after inoculation. At 60 and 90 days after inoculation, there were varying degrees of recovery.

## Discussion

There are at least five *Borrelia* genotypes (*B. burgdorferi* s.s, *B. garinii, B. afzelii*, *B. valaisiana* and *B. sinica*) in China, and *B. burgdorferi* s.s, *B. garinii* and *B. afzelii* have been reported as the main pathogenic genotypes of humans and animals [13]. *B. garinii* is the main genotype in China [9]. *B. garinii* SZ isolated from *Dermacentor* ticks collected in Shangzhi County of Heilongjiang Province in China is very similar to *B. garinii* strain DK27 isolated from Denmark and *B. garinii* Khab isolated from France in terms of the morphology and molecular characteristics [10]. As the presence of *B. burgdorferi* species in *Dermacentor* ticks has been widely noted, but vector competence appears to be very poor [14]. When *B. burgdorferi* B31 low passage strain spirochetes were directly injected into the hemocoel of *Dermacentor variabilis*, the bacteria were cleared early [15]. However, the occasional human cases of Lyme disease in Indiana were observed from *Dermacentor variabilis* ticks [16] and the epidemiology of tick-borne pathogens infecting *Dermacentor* spp. in France has been reported [17]. Hence, they are often omitted from the *Dermacentor* tick investigations. The present study assessed the pathogenicity of *B. garinii* SZ in a murine model.

Evidence from real-time PCR and histopathology indicated that *B. burgdorferi* B31 and *B. garinii* SZ could disseminate to different interior tissues 2 days after infection. This is inconsistent with the previously reported findings in C3H and BALB/c mice infected with *B. burgdorferi* B31, in which the bacteria were initially detected in organs 8–15 days after infection [7,18]. Dissemination of the bacteria as early as 2 days after experimental infection in mice has been reported [19]. This difference can be attributed to the routes of infection, host factors and detection methods of the bacterial burden [20,21]. The presence of *Borrelia* in target tissues and consequent interactions with the host play a role in promoting host inflammation and affect the clinical presentation of disease [12]. Presently, differences in disease severity between *B. burgdorferi* B31 and *B. garinii* SZ infected mice were significant in brain, heart, liver and spleen tissues at 30 days after inoculation. The difference in the clinical manifestation was highly correlated with the difference of the spirochete burden. This phenomenon was also observed by other authors [18]. In B31 infected mice, spirochetes persisted in the kidney. In SZ infected mice, spirochetes persisted in the liver and spleen. These findings might provide a reference for epidemiological investigation of the field samples. The present observation of infections lasting up to 6 months was consistent with prior results [22].

Although bloodstream invasion is an important route, evidence from the real-time PCR indicated that blood spirochete burden in mice infected with the three genospecies of *Borrelia* strains was low. A previous study used a nested and quantitative polymerase chain reaction (qPCR) to detect cell associated *fla*B gene DNA in the plasma of untreated early Lyme disease patients with erythema migrans; the number of *fla*B gene copies did not significantly correlate with any of the clinical, demographic or laboratory variables assessed (8), which may be an important factor in explaining why some spirochetemic patients are completely asymptomatic [23]. However, another study reported that the bacterial burden in blood was significantly correlated with disease severity [12], with the peak number observed 4–7 days after infection. This may explain why some infected patients have only subclinical disease, whereas others develop overt manifestations.

Signs and symptoms of putative failure of BO23 infection in mice or ineffectiveness of infection with BO23 may be formally attributed to the mouse species; it is possible that not all species of mice are susceptible to disseminated infection by *B. burgdorferi* isolates. Variable host susceptibility to different *B. burgdorferi* isolates and complement sensitivity among different Borrelia species has been reported [24].

## Conclusions

The present study aimed to determine the pathogenicity of *B. garinii* SZ, in which *B. burgdorferi* B31 and *B. afzelii* BO23 were used for comparison. The data obtained by real-time PCR and the histopathology indicated the correlations of spirochete burden and disease severity in mouse tissues in infections caused by *B. garinii* SZ, *B. burgdorferi* B31 and *B. afzelii* BO23. The *B. garinii* SZ strain is highly pathogenic and can trigger multi-system pathological damage in mice. Ninety days after infection, the differences in spirochete burden were evident in terms of the persistence and tissue tropism. Infections could persist up to 6 months.

Further studies using experimental infections and transmission with additional clinical isolates may be conducted to analyze vector competency of *Dermacentor spp*. for this pathogen and to clarify the potential role of genotypic variation of *B. burgdorferi* in the pathogenicity of Lyme disease.

## Competing interests

The authors declare that they have no competing interest.

## Authors’ contributions

QW designed the experimental. QW and JW carried out most of the experiments. YL, GG, JY and ZC participated in the design of the study and helped experimental development. QW and ZL drafted the manuscript. JL and HY conceived, coordinated and provided financial support for the study. All authors have read and approved the final version of the manuscript.

## Supplementary Material

Additional file 1: Table S1 MGB-probe based quantitative real time PCR for simultaneous detection and quantification of *B. burgdorferi* in different tissues of BALB/c mice*. Click here for file
