# Supplementary material for: Pathogenic analysis of Borrelia garinii strain SZ isolated from northeastern China
Source: Parasit Vectors. 2013 Jun 17;6:177. doi: 10.1186/1756-3305-6-177 (PMC3689080; doi:10.1186/1756-3305-6-177)
Supplement: Additional file 1: Table S1 — MGB-probe based quantitative real time PCR for simultaneous detection and quantification of B. burgdorferi in different tissues of BALB/c mice*. [file 1756-3305-6-177-S1.doc]

**Supplementary material**

**Table S1. MGB-probe based quantitative real time PCR for simultaneous detection and quantification of *B. burgdorferi* in different tissues of BALB/c mice*.**

| Time p.i. | species | Mouse No. | Brain | tongue | heart | lung | liver | spleen | kidney | lymph | bladder | skin | joint |
| --- | --- | --- | --- | --- | --- | --- | --- | --- | --- | --- | --- | --- | --- |
| 2 d | B31 | 1 | 83,787 | 23,857 | 66,449 | 237,900 | 23,416 | 23,462 | 12,769 | 37,198 | 34,155 | 39,822 | 28,281 |
|  |  | 2 | 83,167 | 23,740 | 63,482 | 105,092 | 25,615 | 24,903 | 21,029 | 30,284 | 17,653 | 40,613 | 25,802 |
|  | SZ | 1 | 20,155 | 33,506 | 50,016 | 61,073 | 8,726 | 2,196 | 35,504 | 33,117 | 28,358 | 71,783 | 9,398 |
|  |  | 2 | 10,195 | 14,161 | 93,140 | 10,811 | 3,328 | 3,122 | 65,907 | 41,688 | 21,315 | 64,146 | 2,676 |
|  | BO23 | 1 | 8 | 116 | 0 | 30 | 37 | 23 | 13 | 0 | 16 | 5 | 26 |
|  |  | 2 | 16 | 80 | 0 | 5 | 12 | 20 | 11 | 4 | 8 | 4 | 15 |
| 5 d | B31 | 1 | 28,013 | 24,195 | 12,295 | 6,152 | 31,857 | 5,543 | 4,886 | 2,508 | 6,361 | 4,038 | 8,078 |
|  |  | 2 | 22,164 | 20,495 | 16,458 | 6,941 | 30,705 | 5,352 | 2,743 | 3,231 | 4,763 | 1,940 | 8,103 |
|  | SZ | 1 | 58,600 | 2,131 | 28,408 | 5,571 | 16,510 | 789 | 2,130 | 193 | 2,499 | 8,581 | 314 |
|  |  | 2 | 68,608 | 3,923 | 21,099 | 5,201 | 15,778 | 770 | 2,612 | 150 | 1,107 | 8,166 | 246 |
|  | BO23 | 1 | 12 | 50 | 0 | 0 | 15 | 20 | 8 | 0 | 0 | 0 | 0 |
|  |  | 2 | 10 | 90 | 0 | 0 | 42 | 35 | 9 | 0 | 0 | 0 | 0 |
| 9 d | B31 | 1 | 1,588 | 1,727 | 264 | 497 | 4,367 | 241 | 920 | 716 | 479 | 1,530 | 393 |
|  |  | 2 | 1,037 | 1,453 | 411 | 676 | 4,364 | 941 | 231 | 216 | 150 | 2,921 | 953 |
|  | SZ | 1 | 48,200 | 24,107 | 5,983 | 12,045 | 318,948 | 15,703 | 34,163 | 8,629 | 7,445 | 16,481 | 8,599 |
|  |  | 2 | 53,262 | 23,285 | 7,001 | 24,962 | 311,246 | 17,797 | 34,177 | 8,464 | 8,534 | 14,612 | 7,625 |
|  | BO23 | 1 | 0 | 0 | 0 | 0 | 0 | 40 | 0 | 80 | 20 | 760 | 90 |
|  |  | 2 | 30 | 0 | 40 | 0 | 40 | 30 | 25 | 79 | 30 | 90 | 120 |
| 15 d | B31 | 1 | 8,683 | 31,508 | 81,412 | 8,984 | 84,444 | 6,251 | 40,471 | 7,575 | 9,440 | 1,507 | 6,600 |
|  |  | 2 | 7,306 | 5,151 | 35,891 | 5,995 | 53,643 | 2,723 | 33,801 | 20,698 | 8,493 | 7,353 | 4,052 |
|  | SZ | 1 | 4,836 | 1,654 | 282 | 299 | 6,308 | 111 | 34 | 103 | 296 | 188 | 95 |
|  |  | 2 | 3,714 | 1,646 | 226 | 220 | 3,458 | 52 | 109 | 163 | 226 | 38 | 38 |
|  | BO23 | 1 | 0 | 0 | 0 | 0 | 0 | 49 | 0 | 90 | 28 | 451 | 41 |
|  |  | 2 | 94 | 0 | 215 | 0 | 17 | 12 | 3 | 0 | 116 | 150 | 2 |
| 30 d | B31 | 1 | 59,794 | 17,104 | 11,485 | 30,142 | 44,961 | 81,109 | 21,957 | 30,176 | 9,186 | 20,589 | 25,571 |
|  |  | 2 | 59,820 | 10,060 | 37,978 | 23,428 | 45,000 | 80,007 | 10,475 | 29,507 | 9,191 | 21,387 | 27,245 |
|  | SZ | 1 | 407,171 | 9,515 | 6,798 | 3,476 | 2,358 | 339 | 113 | 2,129 | 315 | 6,204 | 1,036 |
|  |  | 2 | 417,338 | 9,767 | 7,488 | 2,665 | 3,984 | 448 | 166 | 1,437 | 553 | 6,252 | 1,063 |
|  | BO23 | 1 | 0 | 0 | 45 | 0 | 15 | 36 | 0 | 48 | 52 | 270 | 0 |
|  |  | 2 | 0 | 0 | 25 | 0 | 7 | 48 | 9 | 90 | 110 | 110 | 5 |
| 60 d | B31 | 1 | 379 | 76 | 2,202 | 452 | 375 | 235 | 0 | 47 | 221 | 201 | 93 |
|  |  | 2 | 0 | 330 | 6,870 | 7,503 | 89 | 101 | 54 | 153 | 272 | 119 | 54 |
|  | SZ | 1 | 0 | 0 | 0 | 181 | 596 | 0 | 136 | 134 | 16 | 0 | 0 |
|  |  | 2 | 328 | 0 | 300 | 254 | 1,363 | 81 | 104 | 45 | 244 | 96 | 0 |
| 90 d | B31 | 1 | 0 | 0 | 0 | 0 | 0 | 2,825 | 0 | 0 | 0 | 0 | 0 |
|  |  | 2 | 260 | 75 | 0 | 12 | 0 | 1,025 | 17 | 0 | 0 | 18 | 121 |
|  | SZ | 1 | 0 | 1,349 | 0 | 0 | 4,081 | 616 | 0 | 0 | 0 | 0 | 1,244 |
|  |  | 2 | 11 | 38 | 0 | 75 | 2,508 | 275 | 12 | 0 | 0 | 0 | 174 |
| 150 d | B31 | 1 | 0 | 0 | 0 | 0 | 100 | 276 | 0 | 0 | 0 | 0 | 0 |
|  |  | 2 | 0 | 0 | 0 | 0 | 100 | 321 | 0 | 0 | 0 | 0 | 0 |
|  | SZ | 1 | 0 | 0 | 0 | 97 | 1,200 | 0 | 0 | 0 | 0 | 0 | 0 |
|  |  | 2 | 0 | 0 | 0 | 32 | 815 | 0 | 0 | 0 | 0 | 0 | 0 |

*BALB/c mice were infected with 10^5^ spirochetes intraperitoneally, and were sacrificed by eye bloodletting. The determinations by real-time PCR for individual mice at the indicated times post-infection (p.i.). Two mice from each group were sacrificed at indicated times. Contaminated samples were not included, each sample with three replicates. The numbers of spirochetes per 10^6^ mouse cells was calculated on the basis of*β-actin* standard curve. d, days.
